# Factors for delays in door-to-balloon time ≤ 90 min in an electrocardiogram triage system among patients with ST-segment elevation myocardial infarction: a retrospective study

**DOI:** 10.1186/s12245-023-00562-5

**Published:** 2023-11-02

**Authors:** Atsuhito Inoue, Michiko Mizobe, Jin Takahashi, Hiraku Funakoshi

**Affiliations:** Department of Emergency and Critical Care Medicine, Tokyobay Urayasu Ichikawa Medical Center, 3-4-32 Todaijima, Urayasu, Chiba 279-0001 Japan

**Keywords:** Acute Coronary Syndrome, Chest pain, Quality improvement, ST Elevation Myocardial Infarction

## Abstract

**Background:**

Door to balloon time is a crucial factor of mortality in patients with ST-segment elevation myocardial infarction. However, the factors that contribute to failure of achieving door to balloon time ≤ 90 min in an electrocardiogram triage system remain unknown.

**Methods:**

This single-center retrospective observational study collected data from consecutive patients with ST-segment elevation myocardial infarction from April 2016 to March 2021. The primary outcome was the failure to achieve door to balloon time ≤ 90 min. A multivariate logistic regression model was performed to predict factors associated with failure to achieve door to balloon time ≤ 90 min.

**Results:**

In total, 190 eligible patients were included. Of these, the 139 (73.2%) patients with door to balloon time ≤ 90 min were significantly younger compared to those with door to balloon time > 90 min (*p* = 0.02). However, there was no significant difference in sex and timing of hospital arrival between the door to balloon time ≤ 90 and > 90 min groups. Presence of chest pain and ambulance usage were significantly more frequent in patients with door to balloon time ≤ 90 min (*p* ≤ 0.01, *p* = 0.02, respectively). Multivariate analysis showed that absence of chest pain (adjusted odds ratio 4.76; 95% confidence interval, 2.04–11.1; *p* < 0.01) and non-ambulance usage (adjusted odds ratio 3.53; 95% confidence interval, 1.57–7.94; *p* < 0.01) are predictive factors of failure to achieve door to balloon time ≤ 90 min.

**Conclusion:**

Patients without chest pain as the chief complaint or non-ambulance usage were significantly associated with the failure to achieve door to balloon time ≤ 90 min.

**Supplementary Information:**

The online version contains supplementary material available at 10.1186/s12245-023-00562-5.

## Introduction

Acute coronary syndrome (ACS) is a common disease that is annually diagnosed in more than 7 million people worldwide and has a high mortality rate [1]. ACS is classified into two categories: ST-segment elevation myocardial infarction (STEMI) and non-ST-segment elevation ACS (NSTE-ACS), which consists of non-ST-segment elevation myocardial infarction (NSTEMI) and unstable angina [2, 3]. STEMI accounts for 30–40% of ACS and has higher mortality than NSTE-ACS [4, 5].

Especially for STEMI, early intervention, including primary percutaneous coronary intervention (PCI), is crucial [1]. Many guidelines for ACS recommend a short time interval from the patient's arrival at the hospital to balloon dilatation of the coronary artery (door to balloon time; DTBT) for patients with STEMI [6–8]. Some studies showed that achievement of DTBT ≤ 90 min in patient with STEMI was associated with lower in-hospital mortality than a non-achievement [9, 10], and the American Heart Association advocates DTBT ≤ 90 min as a quality measure in patients with STEMI [11].

To achieve DTBT ≤ 90 min, early diagnosis is essential. Therefore, some clinical guidelines recommend that the time from the patient's arrival at the hospital to performing an electrocardiogram (ECG) (Door to ECG Time; DTET) should be ≤ 10 min if ACS is suspected [3, 7, 8].

Some triage systems, called ECG triage systems, have proposed that ECG should be performed at the time of triage to achieve shorter DTET and DTBT and to prevent oversights [12, 13]. These ECG triage systems have shown high sensitivity for ACS or STEMI and improved the achievement of DTBT ≤ 90 min [14–16].

However, there are limited studies regarding the factors of failure to achieve DTBT ≤ 90 min among the patients with STEMI, with the ECG triage system. Therefore, we conducted a retrospective observational study to predict the factors of failure to achieve DTBT ≤ 90 min among the patients with STEMI with the ECG triage system.

## Methods

### Study design and setting

This was a single-center retrospective observational study, which was conducted at the Emergency department (ED) of a 344-bed urban acute care community hospital in Japan. Annual Emergency room (ER) visits are approximately 25,000 patients including about 10,000 ambulance transportation. This hospital is a 24-h stroke/cardiovascular center with the capability of PCI. The number of ACS patients receiving emergency PCI is about 200 per year. The ECG triage system at this ED requires that ECG is performed within 5 min for all patients with chest pain or palpitations and patients over 45 years of age with epicardial pain, epigastric pain, or nausea without diarrhea and the ECG is reviewed immediately by the attending physician. This ECG triage system was launched in March, 2016 to prevent delays in intervention to a certain number of STEMI patients with the main complaint other than chest pain. Patients transported by ambulance or walk-in are triaged by trained nurses and treated according to triage priorities. Therefore, both patients receive the same medical care. However, patients transported by ambulance may be triaged according to the vital signs reported by the emergency services prior to arrival. The time to triage may therefore be affected.

This study was approved by the ethics committee of the hospital and was conducted according to the ethical guidelines of the Declaration of Helsinki. The ethics committee also approved the waiver of informed consent because of retrospective nature of this study.

### Study participants and population

Myocardial infarction (MI) was defined according to the Fourth Universal Definition of Myocardial Infarction [17]. Diagnostic ST-segment elevation was defined as ST-segment elevation at least two contiguous leads (2.5 mm in men < 40 years, 2 mm in men 40 years, or 1.5 mm in women in leads V 2–V 3 and 1 mm in the other leads). The patients with MI with ST-segment elevation were diagnosed as STEMI [7, 17]. Patients who were diagnosed as STEMI after visiting ED and underwent PCI at this hospital from April 2016 to March 2021 are included. We excluded the following patients: patients who were referred for STEMI by their previous physician, patients who were not initially treated by ER physicians, patients who did not undergo PCI, patients with cardiopulmonary arrest (CPA), and patients who were transferred to other hospitals after diagnosis of STEMI.

### Data collection

We obtained data including age, sex, mode of hospital arrival (ambulance usage or not), the timing of hospital arrival (on-hours or off-hours), symptoms (chief complaint), DTBT, and DTET. On-hours was defined as 9 a.m.-5 p.m. on weekdays. Chief complaint consists of chest pain, palpitations, epicardial pain, epigastric pain, nausea without diarrhea and others. In this study, door time (i.e., the patient's arrival time) was defined as the time of arrival at reception for walk-in patients or the time of ambulance arrival.

### Outcome measure

The primary outcome was the failure to achieve DTBT ≤ 90 min. The secondary outcome was the failure to achieve DTET ≤ 10 min. Both outcomes are defined as clinical indicator in several clinical guidelines [6–8]. In this study, DTBT was defined as the time from the patient's arrival at the hospital to balloon dilatation of the coronary artery. DTET was defined as the time from the patient's arrival at the hospital to performing an ECG.

### Statistical analysis

Continuous variables were expressed as median values with interquartile range (IQR), based on their distributions. Categorical variables were expressed as numbers and percentages. Age, sex, mode of hospital arrival, timing of hospital arrival, chief complaint were compared between the DTBT ≤ 90 min and DTBT > 90 min groups and between the DTET ≤ 10 min and DTET > 10 min group, using the Mann–Whitney U test for continuous variables based on the distributions and the Chi-square test for categorical variables when appropriate. To predict factors associated with failure to achieve DTBT ≤ 90 min and DTET ≤ 10 min, multivariate logistic regression models were constructed, adjusting for the following factors: age, sex, mode of hospital arrival, timing of hospital arrival, and chief complaint.

The statistical analyses were performed using R software (R Core Team, Vienna, Austria). Two-sided *p*-values of < 0.05 were considered statistically significant.

## Results

**Fig. 1:**
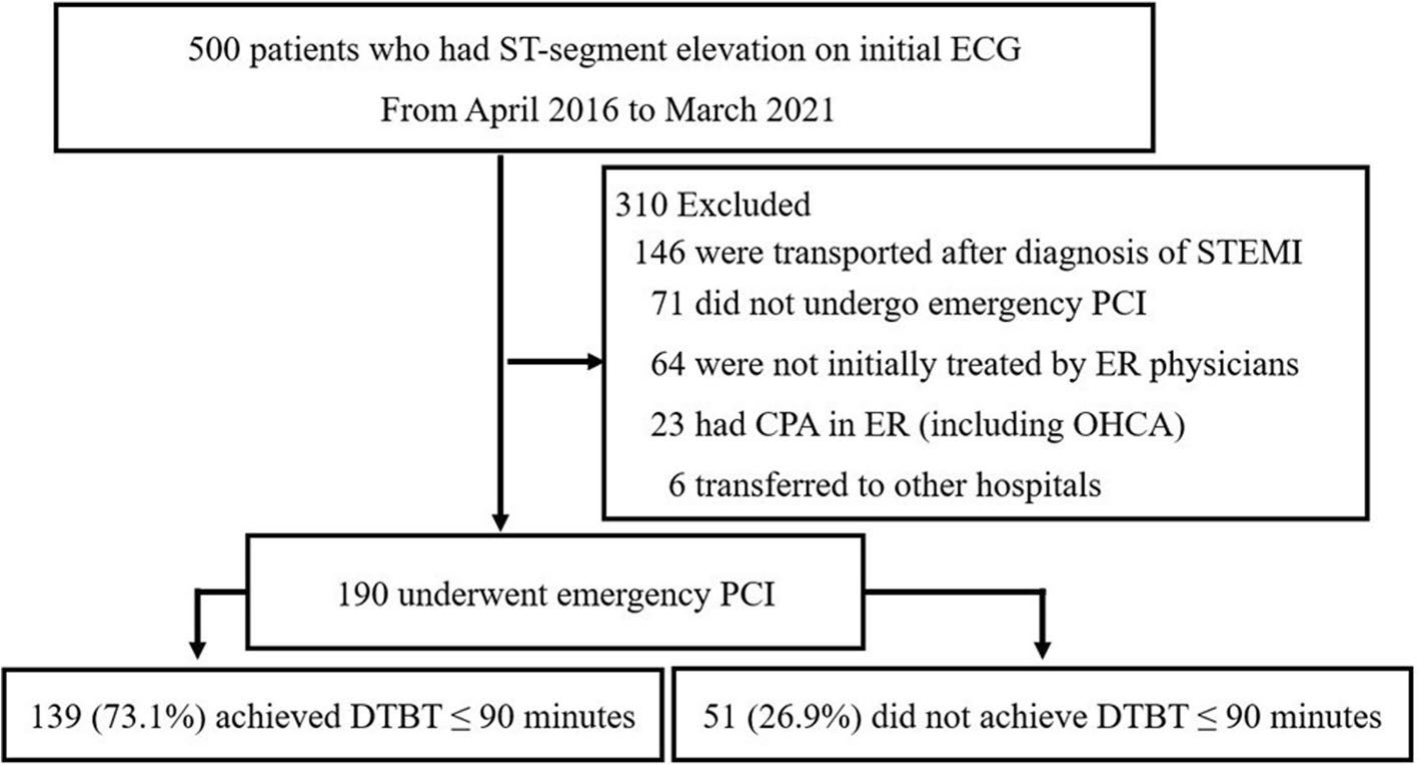
Study flow diagram

Five hundred patients had ST-segment elevation on initial ECG and we excluded 310 patients; 146 were transported after diagnosis of STEMI by the previous doctor, 71 did not undergo emergency PCI, 64 were not initially treated by ER physicians, 23 had CPA (including OHCA) in ER, and 6 were transferred to other hospitals. The remaining 190 patients were eligible for this study (Fig. 1). Patients with DTBT ≤ 90 min were 73.2% (139/190).

The baseline characteristics of each group are shown in Table 1. Patients in DTBT ≤ 90 min group were significantly younger than those in DTBT > 90 min group (the median [IQR] 65 years. [52–74] vs. 72 years [57–82]; *p* = 0.02). Chest pain and ambulance usage were significantly frequent in DTBT ≤ 90 min group than in DTBT > 90 min group (87% vs. 58%; *p* < 0.01, 82% vs. 65%; *p* = 0.02, respectively). There was no significant difference in sex and the timing of hospital arrival between the DTBT ≤ 90 min and DTBT > 90 min groups. Of the 39 patients whose chief complaint was other than chest pain, 9 had dyspnea, 8 had syncope, 6 had nausea without diarrhea, 5 had difficulty moving, 4 had epigastric pain, 3 had upper limb numbness, and 4 had other symptoms.

**Table 1:** Patient characteristics according to door to balloon time

Variables	Overall (*n* = 190)	Door to Balloon Time ≤ 90 min (*n* = 139)	Door to Balloon Time > 90 min (*n* = 51)	*P* value
Age, median (IQR), year	67 (52–77)	65 (52–74)	72 (57–82)	0.02
Male sex	147 (77)	111 (80)	36 (69)	0.18
Non-ambulance usage	44 (23)	26 (18)	18 (35)	0.02
Off-hour presentation^a^	143 (75)	107 (77)	36 (71)	0.45
Absence of chest pain	39 (21)	18 (13)	21 (42)	< 0.01

*Abbreviation*: *IQR* interquartile range

Data were presented as n (%) of patients unless otherwise indicated

Percentages may not equal 100 due to rounding

^a^Off-hour were defined as all other hours

The multivariate regression analysis showed absence of chest pain and non-ambulance usage were predictive factor of failure to achieve DTBT ≤ 90 min (adjusted OR 4.76; 95%CI, 2.04–11.1; *p* < 0.01, adjusted OR 3.53; 95%CI, 1.57–7.94; *p* < 0.01, Table 2).

**Table 2:** Factors associated with failure to achieve Door to Balloon Time ≤ 90 min

Variables	Adjusted odds ratio (95% CI)	*P* value
Age	0.98 (0.95–1.01)	0.16
Male sex	0.92 (0.38–2.21)	0.86
Non-ambulance usage	3.53 (1.57–7.94)	< 0.01
Off-hour presentation	0.98 (0.44–2.17)	0.97
Absence of chest pain	4.76 (2.04–11.1)	< 0.01

*Abbreviation*: *CI* confidence interval

As secondary outcomes, patients with DTET ≤ 10 min were 72.6% (138/190, Appendix Table 1). Multivariate regression analysis showed absence of chest pain and non-ambulance usage were predictive factors of failure to achieve DTET ≤ 10 min (adjusted OR 4.76; 95%CI, 1.92–12.5; *p* < 0.01, adjusted OR 14.5; 95%CI, 5.88–35.8; *p* < 0.01, Table 3).

**Table 3:** Factors associated with failure to achieve Door to ECG Time ≤ 10 min

Variables	Adjusted odds ratio (95%CI)	*P* value
Age	0.97 (0.94–1.00)	0.053
Male sex	1.12 (0.43–2.92)	0.81
Non-ambulance usage	14.5 (5.88–35.8)	< 0.01
Off-hour presentation	1.06 (0.45–2.50)	0.89
Absence of chest pain	4.76 (1.92–12.5)	< 0.01

*Abbreviation*: *CI* confidence interval

## Discussion

This study showed that absence of chest pain and non-ambulance usage were predictive factors for the failure to achieve DTBT ≤ 90 min. On the other hand, off-hours presentation was not a predictive factor.

The absence of chest pain as the chief complaint was associated with a significantly higher rate of failure to achieve DTBT ≤ 90 min and DTET ≤ 10 min. Consistent with our results, previous studies have reported that the absence of chest pain was an independent risk factor for delayed DTBT [18, 19]. Our results suggested that the absence of chest pain could delay the diagnosis and intervention of STEMI because absence of chest pain makes it harder for healthcare providers to suspect STEMI. Triage providers need to triage high-risk patients even in the absence of chest pain.

Non-ambulance usage was associated with a significantly higher rate of failure to achieve DTBT ≤ 90 min. Previous studies reported that arrival by walk-in was an independent factor for the failure to achieve DTBT ≤ 90 min similarly [20, 21]. In addition, our study also showed an association of non-ambulance usage with a significantly higher rate of failure to achieve DTET ≤ 10 min. Therefore, we believe that establishing a rapid ECG implementation system after arrival at the hospital by walk-in is necessary.

In this study, off-hours presentation was not associated with the failure to achieve DTBT ≤ 90 min. This was contrary to previous studies, which reported that off-hours presentation was significantly associated with a higher rate of failure to achieve DTBT ≤ 90 min and of in-hospital mortality [22]. Holmes et al. reported the usefulness of STEMI protocol during off hours and emphasized the introduction of STEMI protocols [23]. Since no off-hours delays were observed in this study, the introduction of the ECG triage system could have contribution to reducing off-hours DTBT.

In this study, the absence of chest pain was still a predictive factor for the failure to achieve DTET ≤ 10 min despite the ECG triage system included symptoms other than chest pain to the indication of ECG. The primary cause could be human factors such as individual experience and cognitive biases in the triage system. The previous study reported that more experienced ER nurses tend to perform under-triage during triage assignment [24]. In addition, many ER triage nurses in the experienced facility tend to perform triage ECG based on their own clinical assessment rather than the triage guideline in the facility [25]. Therefore, continuous monitoring and feedback could be essential for compliance with triage protocol enforcement.

This study revealed that the factors for delayed diagnosis and treatment of STEMI patients is absence of chest pain or non-ambulance usage. The advance triage system that performs ECGs, even if patients have no chest pain or did not arrive by ambulance, could potentially prevent delays in diagnosis and treatment of STEMI. However, comprehensive testing could increase medical costs. Therefore, further research is warranted to verify the cost-effectiveness of such a system. As this research, exploratory research after the introduction of triage systems can lead to the development of a standardized Triage ECG System. The standardized triage system will be validated in a multicenter and prospective study.

Our study had several potential limitations. First, our study was a retrospective observational study conducted at a single facility with urban ER-based emergencies. Generalizability may not be applicable. Second, potentially unmeasured confounders affected DTBT or DTET, such as vital signs at arrival and co-morbid conditions. Third, the accuracy of the medical records may have been hampered by vague symptom descriptions by the physicians in charge.

## Conclusion

Patients without chest pain as the chief complaint or non-ambulance usage were significantly associated with the failure to achieve DTBT ≤ 90 min and DTET ≤ 10 min.

### Supplementary Information

**Additional file 1:** **Appendix Table 1.** Patient characteristics according to door to ECG time.

## Data Availability

Due to the nature of this research, participants of this study did not agree with the sharing of their data publicly; supporting data are unavailable.
