# Supplementary material for: Factors for delays in door-to-balloon time ≤ 90 min in an electrocardiogram triage system among patients with ST-segment elevation myocardial infarction: a retrospective study
Source: Int J Emerg Med. 2023 Nov 2;16:77. doi: 10.1186/s12245-023-00562-5 (PMC10621087; doi:10.1186/s12245-023-00562-5)
Supplement: Supplementary file 1 — Additional file 1: Appendix Table 1. Patient characteristics according to door to ECG time. [file 12245_2023_562_MOESM1_ESM.docx]

Appendix Table 1. Patient characteristics according to door to ECG time

| Variables | Overall  (n=190) | Door to ECG Time ≤ 10minutes (n=138) | Door to ECG Time > 10minutes (n=52) | P value |
| --- | --- | --- | --- | --- |
| Age, median (IQR), year | 67 (52-77) | 65 (57-82) | 69 (57-82) | 0.04 |
| Male sex | 147 (77) | 109 (79) | 38 (73) | 0.43 |
| Non-ambulance usage | 44 (23) | 17 (12) | 27 (52) | <0.01 |
| Off-hour presentation^*^ | 143 (75) | 107 (77) | 36 (69) | 0.26 |
| Absence of chest pain | 39 (21) | 20(14) | 19 (37) | <0.01 |

Abbreviation: IQR, interquartile range.

Data were presented as n (%) of patients unless otherwise indicated.

Percentages may not equal 100 due to rounding.

*Off-hour were defined as all other hours.
